# Glibenclamide pretreatment attenuates early hematoma expansion of warfarin-associated intracerebral hemorrhage in rats by alleviating perihematomal blood–brain barrier dysfunction

**DOI:** 10.1186/s41016-023-00351-2

**Published:** 2023-12-07

**Authors:** Zongwei Zeng, Liang Liang, Zhou Feng, Peiwen Guo, Xiaoke Hao, Jishu Xian, Hua Feng, Yujie Chen, Zhi Chen

**Affiliations:** 1 Department of Neurosurgery and State Key Laboratory of Trauma, Burn and Combined Injury, Southwest Hospital, Third Military Medical University (Army Medical University), Chongqing, 400038 China; 2 Department of Neurosurgery, The 945Th Hospital of PLA, Yaan, Sichuan Province 625099 China; 3 Department of Rehabilitation, Southwest Hospital, Third Military Medical University (Army Medical University), Chongqing, 400038 China; 4 Chongqing Key Laboratory of Precision Neuromedicine and Neuroregeneration, Southwest Hospital, Third Military Medical University (Army Medical University), Chongqing, 400038 China; 5 Chongqing Clinical Research Center for Neurosurgery, Southwest Hospital, Third Military Medical University (Army Medical University), Chongqing, 400038 China

**Keywords:** Intracerebral hemorrhage, Warfarin, Glibenclamide, Hematoma expansion, Blood–brain barrier, Matrix metallopeptidase-9

## Abstract

**Background:**

Hematoma expansion is a determinant of poor outcome of intracerebral hemorrhage but occurs frequently, especially in warfarin-associated intracerebral hemorrhage (W-ICH). In the present study, we employ the warfarin-associated intracerebral hemorrhage (W-ICH) rat model, to explore the efficacy and potential mechanism of glibenclamide pretreatment on hematoma expansion after intracerebral hemorrhage, hoping to provide proof of concept that glibenclamide in stroke primary and secondary prevention is also potentially beneficial for intracerebral hemorrhage patients at early stage.

**Methods:**

In the present study, we tested whether glibenclamide, a common hypoglycemic drug, could attenuate hematoma expansion in a rat model of W-ICH. Hematoma expansion was evaluated using magnetic resonance imaging; brain injury was evaluated by brain edema and neuronal death; and functional outcome was evaluated by neurological scores. Then blood–brain barrier integrity was assessed using Evans blue extravasation and tight junction-related protein.

**Results:**

The data indicated that glibenclamide pretreatment significantly attenuated hematoma expansion at 24 h after W-ICH, thus mitigating brain edema and neuronal death and promoting neurological function recovery, which may benefit from alleviating blood–brain barrier disruption by suppressing matrix metallopeptidase-9.

**Conclusions:**

The results indicate that glibenclamide pretreatment in stroke primary and secondary prevention might be a promising therapy for hematoma expansion at the early stage of W-ICH.

**Supplementary Information:**

The online version contains supplementary material available at 10.1186/s41016-023-00351-2.

## Background

Intracerebral hemorrhage (ICH) is the deadliest type of stroke that lacks proven scientific treatments [1]. For a long time, ICH was considered beyond intervention as clinicians believed the hematoma formed over minutes. It was not until the observation of hematoma expansion after ICH that researchers realized a new direction, which recently been frequently found in the first 2 h after ICH by post hoc analysis of BEST-MSU multicenter study (Trial No. NCT0190500) [2]. As the volume of hematoma is considered to be the major determinant of the prognosis of ICH, preventing hematoma expansion has been regarded as a promising therapeutic strategy [3].

Conspicuously, hematoma expansion was reported to occur more frequently in anticoagulation-associated ICH, a severe subtype of ICH that occurs during anticoagulation that is associated with higher short-term mortality and worse clinical outcome, than nonanticoagulated spontaneous ICH [4]. What is more, the incidence of anticoagulation-associated ICH increases significantly as the population ages [5]. Unfortunately, there are still no effective interventions though we are aware of these high-risk people [6]. Yan Z. et al. recently performed a meta-analysis of the function of tranexamic acid to prevent expansion after ICH, the data indicated has tranexamic acid could prevent hematoma expansion, unfortunately without the efficacy of improving neurological prognosis [7].

Glibenclamide (GLC) is a widely used drug for the treatment of type 2 diabetes mellitus and stroke primary or secondary prevention, which belongs to the sulfonylurea class. Our previous study indicated GLC has pleiotropic protection for aged ICH rats [8]. Even more strikingly, recent studies about diabetic patients with ICH indicated that patients with sulfonylureas pretreatment had significantly lower hematoma volumes and better outcomes [9]. In addition, Zhao J. et al. demonstrated GLC therapy could attenuate peri-hematoma edema but without significantly reducing the proportion of poor outcomes for ICH patients (GATE-ICH trial, No. NCT03741530) [10]. Thus, these findings prompt us to speculate whether sulfonylureas pretreatment might provide a preventive intervention for the high-risk population of intracerebral hemorrhage with oral anticoagulants.

In the present study, we employ the warfarin-associated intracerebral hemorrhage (W-ICH) rat model, to explore the efficacy and potential mechanism of GLC pretreatment on hematoma expansion after ICH, hoping to provide proof of concept that GLC in stroke primary and secondary prevention might be a promising therapy for hematoma expansion at the early stage of W-ICH.

## Methods

### Animals and groups

One hundred and forty (140) adult male Sprague–Dawley rats (250–300 g, 8 weeks old; Experimental Animal Center of Army Medical University) were used for the entire study. All experiments are reported in compliance with the Animal Research: Reporting in vivo Experiments version 2.0 (ARRIVE 2.0) guidelines. The experimental protocols were approved by the Laboratory Animal Welfare and Ethics Committee of Army Medical University (AMUWEC2020917) and performed according to the Guide for the Care and Use of Laboratory Animals. Animals were maintained under standard pathogen-free conditions (23–25 ℃, 70% humidity, 12 h light/dark cycle).

Our experiment consists of 2 parts. In the 1st part, animals were randomly divided into sham-operated group (sham group, *n* = 6), ICH (ICH group *n* = 6), and warfarin pretreatment + ICH (W-ICH group *n* = 6). All animals underwent magnetic resonance imaging (MRI) examinations at 24 h after ICH. In the 2nd part, animals were randomly divided into sham-operated group (sham group), ICH with vehicle treatment (vehicle group), and ICH with glibenclamide treatment (GLC group) after warfarin treatment. Some animals (*n* = 6/group) underwent MRI examinations at 24 h after ICH and were euthanized for hemoglobin assay. Some animals (*n* = 6/group) underwent functional assessment at 24 h after ICH and then were euthanized for brain water content (BWC) measurement. The remaining animals were used for Evans blue (EB) fluorescence (*n* = 6 per group) and quantification (*n* = 6/group), immunofluorescence (*n* = 6/group), and Western blot (WB, *n* = 6/group).

### Warfarin administration and INR measurement

Warfarin pretreatment was performed as described [11]. Briefly, warfarin (warfarin sodium; Bristol Myers Squibb) was dissolved in distilled water. A 0.2 mg/kg of warfarin was administered by oral uptake during 24 h, which began 24 h prior to ICH.

International normalized ratio (INR) measurement was performed on a routine analyzer platform in the Southwest Hospital Affiliated to Army Medical University. After warfarin pretreatment, the mean INR value of treated animals was 3.0 ± 0.4, which is similar to the therapeutic range in humans [11].

### Glibenclamide pretreatment therapy

GLC (Sigma) was administrated as described [12]. After dissolved in dimethyl sulfoxide (DMSO), GLC was diluted with saline and sodium hydroxide (NaOH, final pH ~ 8.5) to 1 or 200 μg/ml. Initially, a loading dose of GLC (10 ug/kg) was administrated intraperitoneally 24 h before ICH. Then, GLC was continuously administrated (200 ng/h) by a mini osmotic pump (1.0 μl/h; Alzet Corp) subcutaneously until animals were executed. The vehicle group received the same dose of solution (made with corresponding concentrations of DMSO, saline, and NaOH) by the same route of administration.

### Surgical procedures

The collagenase-induced ICH was made according to previous methods with some modifications [13]. Briefly, after anesthetized using isoflurane (1.5 to 2%) with a nitrous oxide/oxygen mixture, rats were fixed to a stereotaxic frame. After a cranial bur hole (coordinates: 0.2 mm anterior and 3.5 mm lateral to the bregma) 1 mm in diameter was drilled, a microinjector was inserted into the right striatum (6.0 mm deep from the dura) and 1.0 μl of saline containing 0.1 U of Collagenase IV (Sigma, USA) was infused over 5 min. After being left in place for 5 min, the microinjector was slowly removed. Finally, the burr hole was sealed with bone wax. The sham group underwent only needle injection.

### MRI examination and hematoma volume measurement

MRI examination was performed 24 h after ICH induction using a 7.0 T Varian MR scanner (Bruker) [14]. After animals were anesthetized (2% isoflurane/air mixture), coronal T2* gradient-echo and T2-weighted images were obtained by viewing a field of 35 mm × 35 mm from each animal. Hematoma volume was calculated using T2* gradient-echo images as previously described [14]. Briefly, the area of hematoma was outlined in each slice and measured by Image J (National Institutes of Health). Then, the volume was calculated by the total areas of all slices multiplied by the section thickness (1 mm). Brain edema was evaluated by brain swelling (%) using T2-weighted images calculated by the previously reported formula [15]: brain swelling (%) = (ipsilateral hemisphere − contralateral hemisphere)/contralateral hemisphere × 100%. All image analyses were performed independently by two observers blinded to the animal groups.

### Blood volume evaluation (hemoglobin assay)

Intracerebral bleed volume was evaluated using a modified spectrophotometric assay reported previously with some modifications [16]. Briefly, after deeply anesthetized, animals were perfused transcardially with saline at 24 h after ICH. Then ipsilateral hemispheres were collected to homogenate in distilled water (3 mL) for 60 s, followed by centrifuging at 13,000 g for 30 min. Equal amounts of supernatant (100 μL) were obtained to mix with Drabkin’s reagent (400 μL, Sigma) for 15 min at room temperature. The absorbance was measured at 540 nm using a spectrophotometer, and the amount of hemoglobin was determined based on a standard curve generated as described previously [16]. The results were presented as μL of blood in the ipsilateral hemisphere.

### Brain water content

Brain water content measurement was performed at 24 h after ICH. Animals were decapitated after being deeply anesthetized. Then brains were immediately removed and divided into five parts: ipsilateral cortex (Ipsi-CX), ipsilateral basal ganglia (Ipsi-BG), contralateral cortex (Contra-CX), contralateral basal ganglia (Contra-BG), and cerebellum (Cerebel; used as internal control). Brain tissues were weighed immediately after cutting. After drying for 24 h at 100 ℃, brain tissues were weighed again. The BWC (%) = (wet weight − dry weight)/wet weight × 100%.

### Neurological function assessment

The neurological function were assessed at 24 h after ICH by the following three tests [13]: (1) The mNSS, the mNSS (normal: 0; maximal deficit score: 18) was used to comprehensively evaluates sensory, motor, and balance functions. (2) The forelimb placing test (%), the percentage of whether the animal placed the appropriate forelimb to the countertop after 10 times of whisker stimulation was calculated. (3) The corner turn test, the percentage of right turns when the animal exited a 30° corner was recorded as the score (%). All tests were evaluated blindly by two experimenters.

### Evans blue (EB) assay

EB extravasation was performed at 24 h after ICH as reported previously [13]. For EB fluorescence, EB solution (2% w/v; 4 mL/kg, Sigma) was injected intravenously. Then 1 h later, animals were deeply anesthetized, and the brains were removed immediately. After being fixed in 4% paraformaldehyde overnight and dehydrated in gradient concentration sucrose, brains were cut into 18-μm-thick slices. Finally, slices were examined using a confocal microscope (LSM880, Zeiss).

For EB quantification, animals were anesthetized and perfused using a phosphate-buffered solution (PBS) after the EB solution was injected. Then brains were harvested and weighed rapidly, followed by homogenized in PBS. After being centrifuged at 15,000 g for 30 min, the supernatants were obtained. Samples were mixed with the same volumes of trichloroacetic acid (Sigma) and incubated at 4 °C overnight. Then supernatants were obtained after being centrifuged at 15,000 g for 35 min, and the content of EB was determined by spectrophotometry at wavelength of 620 nm. The results were expressed as EB/brain (μg/g).

### Immunofluorescence

Immunofluorescence was performed according to the previous method [17]. Animals were deeply anesthetized and perfused with PBS and 4% paraformaldehyde sequentially. Then brains were removed and fixed in 4% paraformaldehyde overnight. After being dehydrated with gradient concentration sucrose, brains were cut coronally into 18-μm tissue sections. The sections were washed with PBS, permeabilized by Triton X-100, and blocked with 5% BSA for 1 h. Then the sections were incubated with primary antibodies overnight at 4 °C and appropriate secondary antibodies for 1.5 h at 37 °C sequentially. Finally, the sections were incubated with DAPI to stain nuclei and observed under the confocal microscope (LSM880, Zeiss) or fluorescence microscope (AX10 IMAGER.A2, Zeiss). The used primary antibodies are listed: rabbit anti-ZO-1 antibody (1:300, Thermo Fisher); mouse anti-von Willebrand factor (vWF) antibody (1:200, Santa Cruz); rabbit anti-occludin antibody (1:100, Abcam); goat anti-Iba-1 antibody (1:200, Abcam); and rabbit anti-matrix metallopeptidase-9 (MMP-9) antibody (1:300, Abcam).

### TUNEL staining

Brain samples were permeabilized by Triton X-100 and incubated with 5% BSA for 1 h. Then samples were incubated with rabbit anti-NeuN (1:500, CST) primary antibody overnight at 4 °C and appropriate secondary antibody for 1.5 h at 37 °C sequentially. After incubated with a TUNEL reaction mixture (In Situ Cell Death Detection Kit, TMR red; Roche) for 1 h at 37 °C, samples were stained by DAPI. Finally, samples were examined under the confocal microscope (LSM880, Zeiss). The number of TUNEL-positive cells and the proportion of TUNEL-positive neurons were evaluated using Image J.

### Western blot

Western blot (WB) was performed according to previous methods [18]. Briefly, after animals were deeply anesthetized and perfused transcardially with saline, perihematomal brain tissues (4 mm thick) were removed immediately on ice. The obtained brain tissues were lysed by RIPA buffer containing protease and phosphatase inhibitors and centrifuged at 13,000 g for 20 min. Then, protein concentrations were measured using a BCA Protein Quantitation Kit (Beyotime). Equal protein samples (30 μg) were loaded into the SDS-PAGE gel to separate and transfer onto PVDF membranes. After being blocked, membranes were incubated with primary antibodies at 4 °C overnight, followed by incubating in the appropriate HRP-conjugated secondary antibody for 2 h at room temperature. Finally, the bands were visualized under Western Lightning-ECL (Bio-Rad, USA) and analyzed using Image J. The used primary antibodies are listed: rabbit anti-ZO-1 (1:1000, Thermo Fisher); rabbit and anti-Occludin (1:1000, Abcam); rabbit anti-MMP9 antibody (1:1000, Abcam).

### Statistical analysis

All statistical analysis was done by GraphPad Prism 8.0. Comparisons between the two were performed using an unpaired Student’s *t*-test. Multigroup comparisons were performed using a one-way analysis of variance (ANOVA) followed by the Bonferroni post hoc test. Data were expressed as means ± standard deviation. And *P* < 0.05 was considered statistically significant.

## Results

### Effect of GLC on blood glucose

Blood glucose concentration was evaluated at different time points. The blood glucose level of the GLC group exhibited no significant difference compared with that of the vehicle group at all time points (Supplemental Table 1). This indicates that the dose of GLC used in the present study does not impact blood glucose.

### Warfarin increased hematoma volume after ICH induction

**Fig. 1:**
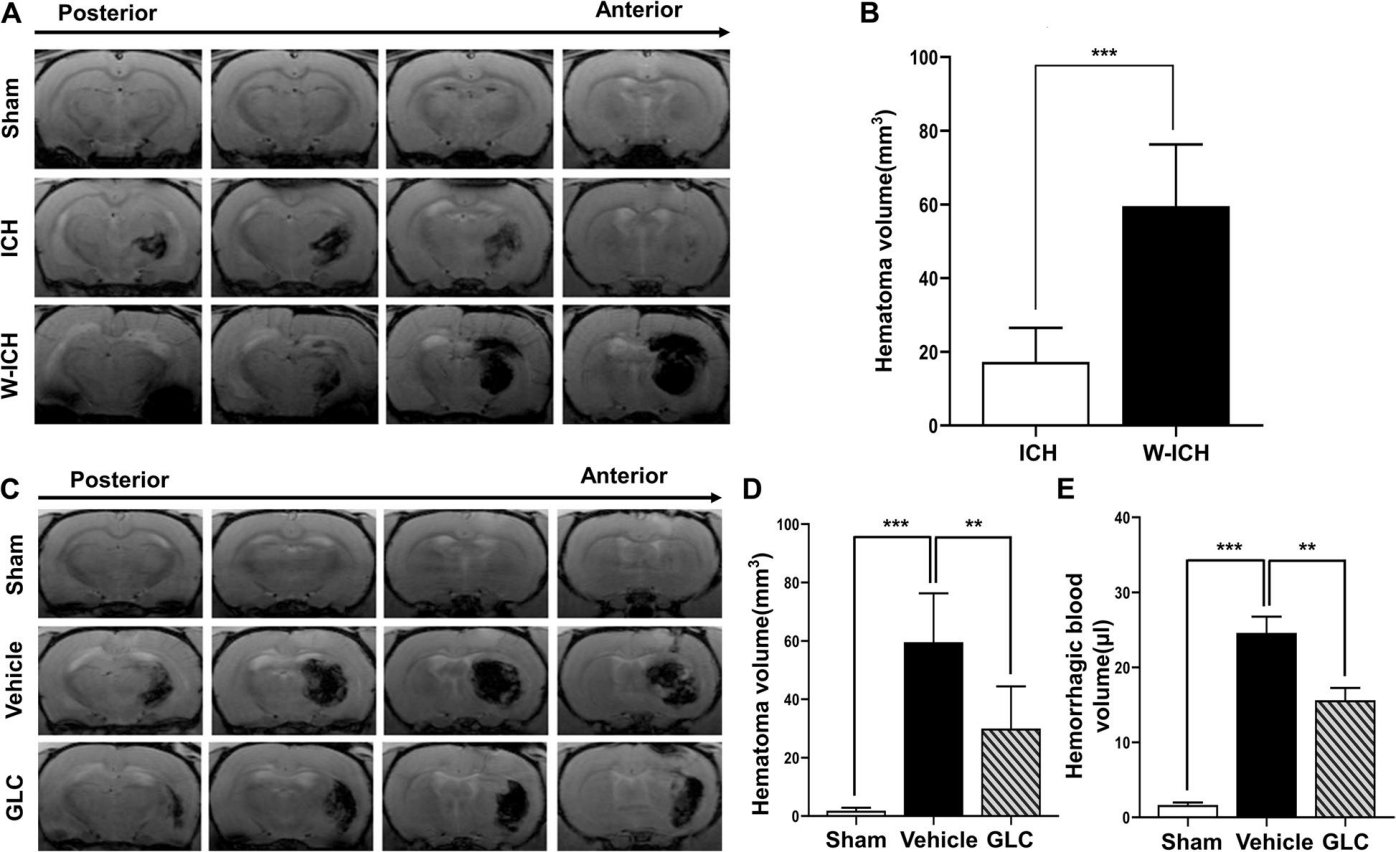
GLC pretreatment attenuated hematoma expansion of W-ICH. **A** Representative T2* gradient-echo images from each group at 24 h after ICH. **B** Quantification of hematoma volume according to T2* gradient-echo images from each group (*n* = 6). **C** Representative T2* gradient-echo images from each group at 24 h after W-ICH. **D** Quantification of hematoma volume according to T2* gradient-echo images from each group (*n* = 6). **E** Quantification of intracerebral bleed volume hemoglobin assay (*n* = 6). Data are presented as mean ± SD. **P < 0.01; ***P < 0.001

T2* gradient-echo images were obtained at 24 h after ICH to evaluate hematoma volumes of each group (Fig. 1A). Warfarin treated significantly increased hematoma volume after ICH compared with normal ICH (59.57 ± 16.72 mm^3^ vs 17.25 ± 9.28 mm^3^, *P* < 0.001; Fig. 1B). This finding suggests that warfarin aggravates hematoma expansion after ICH.

### GLC pretreatment attenuated hematoma expansion in W-ICH

T2* gradient-echo images were obtained at 24 h after ICH to evaluate hematoma volumes of each group (Fig. 1C). GLC pretreatment significantly reduced hematoma volume compared with vehicle treatment in W-ICH (29.97 ± 14.45 mm^3^ vs 58.97 ± 16.27 mm^3^, *P* < 0.01; Fig. 1D). It shows that GLC attenuated hematoma expansion in W-ICH.

Intracerebral bleed volume was analyzed by hemoglobin assay at 24 h after ICH. In agreement with hematoma volume, GLC pretreatment significantly reduced bleed volume compared with vehicle treatment in W-ICH (15.62 ± 1.65 μl vs 24.60 ± 2.17 μl, *P* < 0.01; Fig. 1E).

### GLC pretreatment ameliorated brain edema in W-ICH

**Fig. 2:**
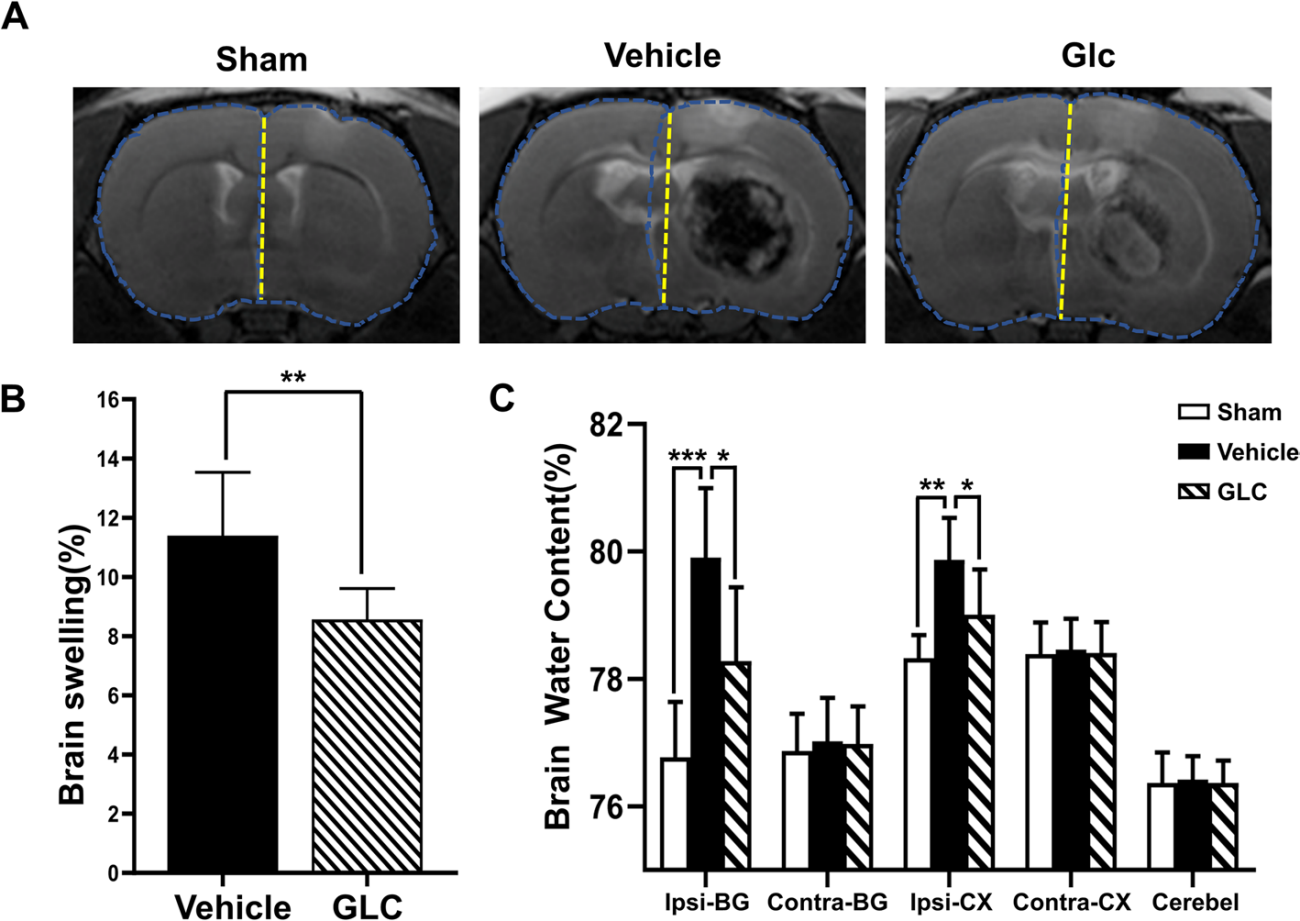
GLC pretreatment ameliorated brain edema in W-ICH. **A** Representative T2-weighted images from each group at 24 h after W-ICH. **B** Quantification of brain swelling (%) according to the related T2-weighted images (*n* = 6). **C** Brain water content determination of ipsilateral basal ganglia (Ipsi-BG), ipsilateral cortex (Ipsi-CX), contralateral basal ganglia (Cont-BG), contralateral cortex (Cont-CX), and cerebellum (Cerebel) (*n* = 6). Data are shown as mean ± SD. *P < 0.05; **P < 0.01; ***P < 0.001

T2-weighted images obtained at 24 h after ICH were used to assess brain edema (Fig. 2A). Brain swelling (%) quantified by T2-weighted images showed that GLC pretreatment significantly alleviated brain swelling compared with vehicle group (8.57 ± 1.05% vs 11.40 ± 2.13%, *P* < 0.01) after W-ICH (Fig. 2B). Further, brain edema was evaluated by brain water content at 24 h after W-ICH. Similarly, compared to the sham group, the water content of ipsilateral basal ganglia increased after W-ICH (76.77 ± 0.87% vs 79.90 ± 1.09%, *P* < 0.001), which was reduced effectively by GLC pretreatment (GLC 78.28 ± 1.16% vs vehicle 79.90 ± 1.09%, *P* < 0.05; Fig. 2C). These findings suggests that GLC pretreatment ameliorated brain edema in W-ICH.

### GLC pretreatment reduced cell death following W-ICH

**Fig. 3:**
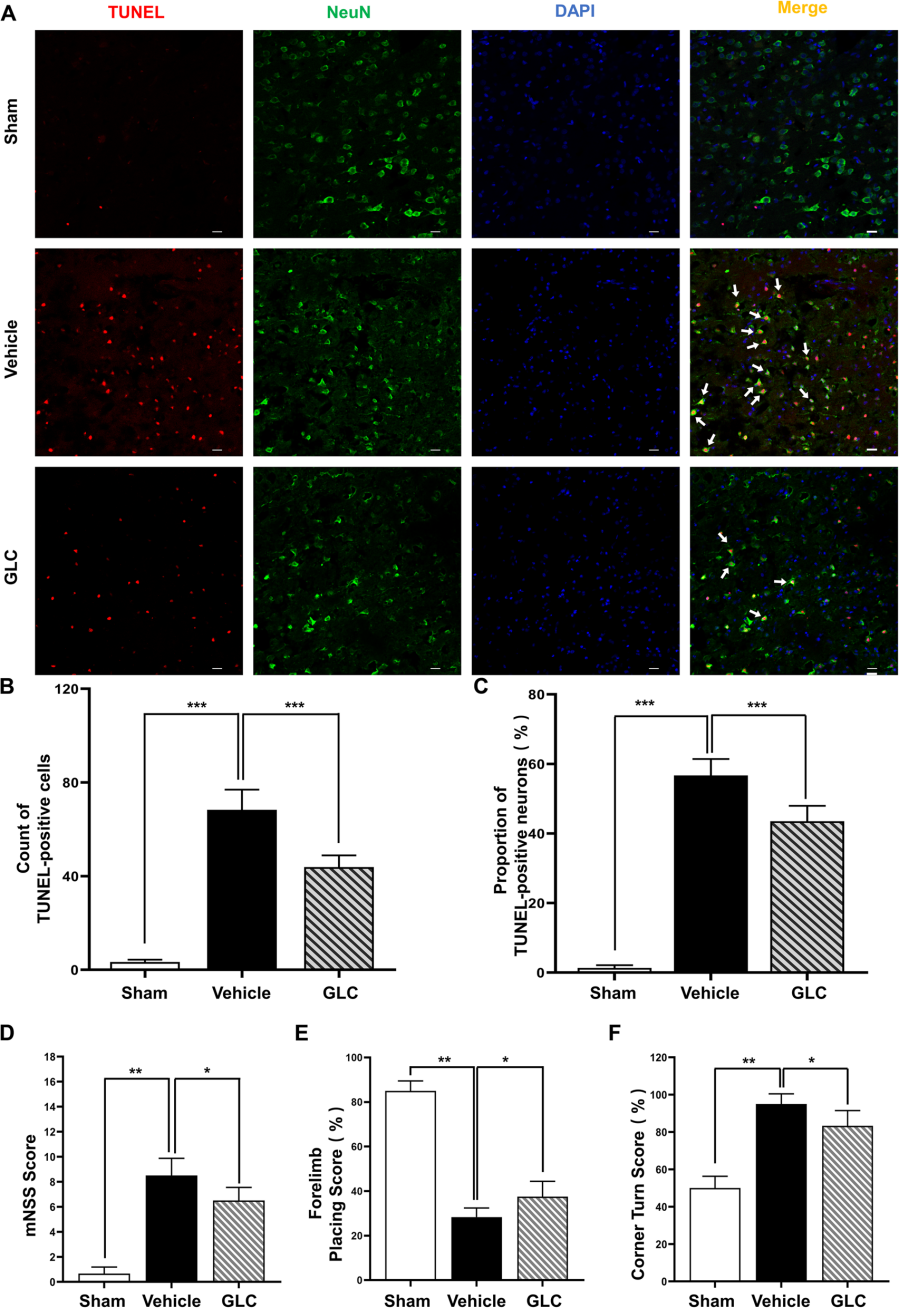
GLC pretreatment attenuated neurological deficits following W-ICH. **A** Representative confocal images of TUNEL-positive neurons in the perihematomal area. **B** Counting of total TUNEL-positive cells. **C** Analysis of the proportion of TUNEL-positive neurons. Results of the mNSS score (**D**), the forelimb placing score (**E**), and the corner turn test (**F**) at 24 h after W-ICH (*n* = 6). Scale bar = 20 µm. Data are shown as mean ± SD. *P < 0.05; **P < 0.01; ***P < 0.001

TUNEL staining was performed to assess perihematomal cell death following W-ICH. W-ICH induced massive cell death at 24 h following surgery (sham 3.33 ± 1.03 vs vehicle 68.33 ± 8.64, *P* < 0.001; Fig. 3A–C), which was significantly suppressed by GLC pretreatment (GLC 43.83 ± 5.04 vs vehicle 68.33 ± 8.64, *P* < 0.001; Fig. 3A–C). In addition, NeuN (neuronal maker) co-staining with TUNEL was performed to evaluate perihematomal neuronal cell death. The proportion of TUNEL-positive neurons in the vehicle group increased significantly after W-ICH (sham 1.33 ± 0.82% vs vehicle 56.67 ± 4.76%, *P* < 0.001; Fig. 3A–C), while GLC pretreatment ameliorated neuronal cell death (GLC 43.50 ± 4.46% vs vehicle 56.67 ± 4.76%, *P* < 0.001; Fig. 3A–C). The above results indicate that GLC pretreatment suppressed neuronal cell death following W-ICH.

### GLC pretreatment attenuated neurological deficits following W-ICH

The neurological function was assessed using the mNSS score, forelimb placing score, and corner turn test at 24 h after W-ICH. W-ICH led to significant neurological deficits (sham vs vehicle, *P* < 0.01; Fig. 3D–F), which was improved by GLC pretreatment (GLC vs vehicle, *P* < 0.05; Fig. 3D–F).

### GLC pretreatment reduced EB extravasation following W-ICH

**Fig. 4:**
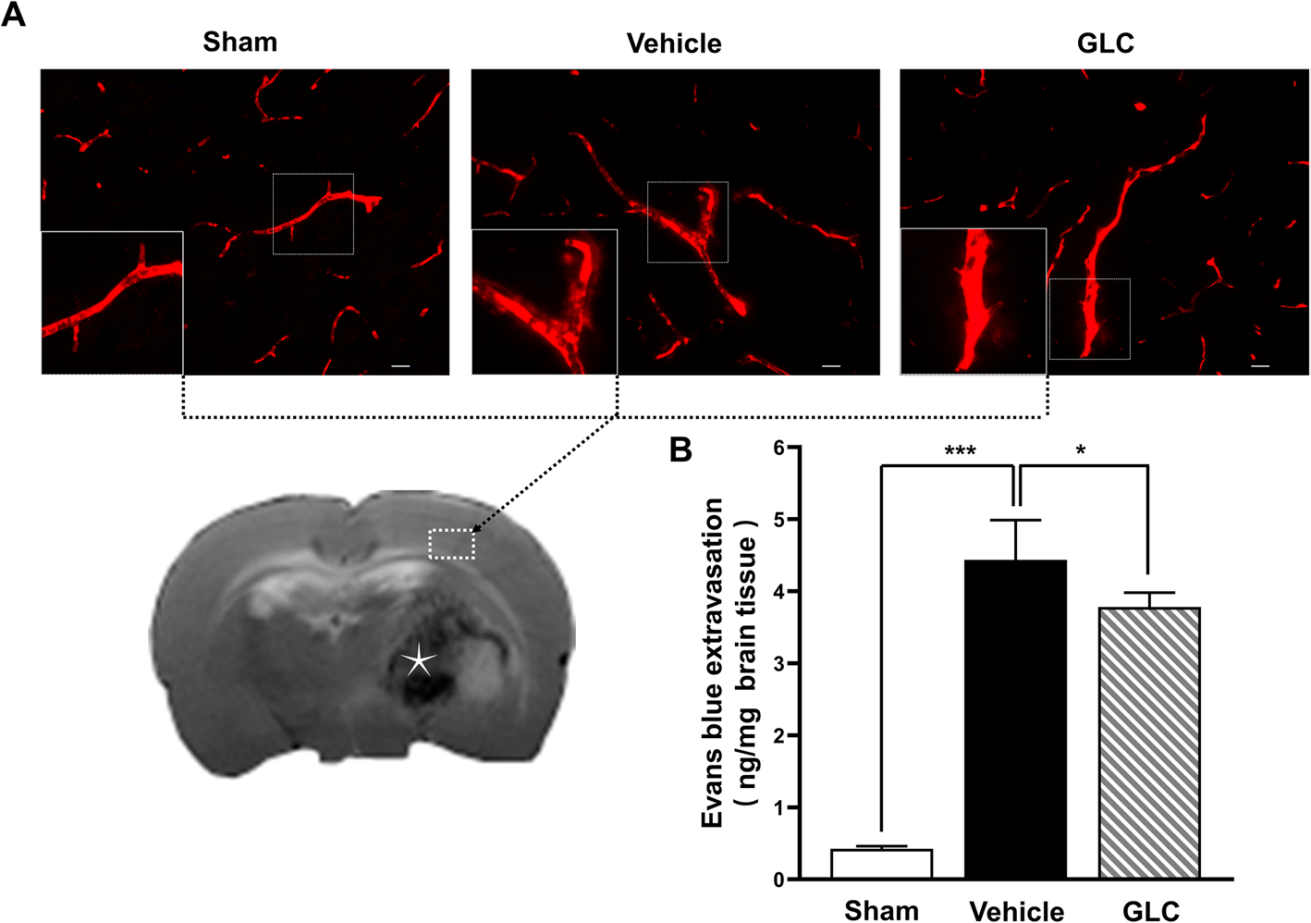
GLC pretreatment reduced EB extravasation following W-ICH. **A** Representative confocal images display the perivascular EB dye leakage from vessels of the ipsilateral cortex. An asterisk in the brain section indicates the intracerebral hematoma. Scale bar = 20 µm. **B** Quantification of perivascular EB extravasation at 24 h after W-ICH (*n* = 6). Data are shown as mean ± SD. *P < 0.05; ***P < 0.001

EB extravasation was used to evaluate the blood–brain barrier (BBB) integrity at 24 h after W-ICH. EB fluorescence images showed obvious EB extravasation in the vehicle group, but decreased by GLC pretreatment (Fig. 4A). EB quantification further indicated that W-ICH led to Evans blue leakage after W-ICH (sham 0.42 ± 0.38 vs vehicle 4.44 ± 0.55, *P* < 0.001). However, GLC pretreatment significantly reduced Evans blue leakage (GLC 3.78 ± 1.99 vs vehicle 4.44 ± 0.55, *P* < 0.05; Fig. 4B). It suggests that GLC pretreatment protected blood–brain barrier (BBB) integrity after W-ICH.

### GLC pretreatment prevented BBB destruction following W-ICH

**Fig. 5:**
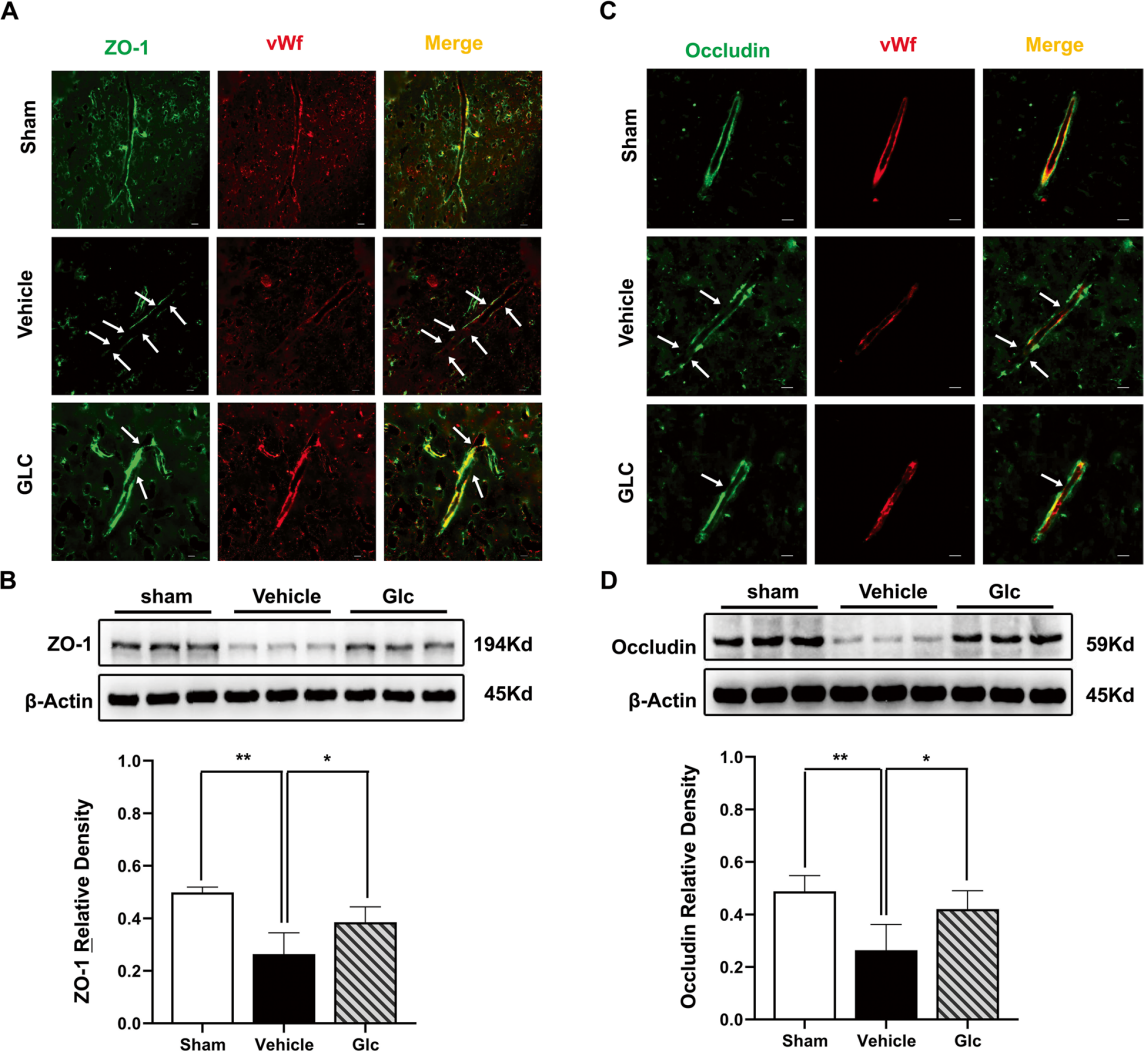
GLC pretreatment prevented BBB destruction following W-ICH. Representative confocal images of BBB tight junction protein ZO-1 (**A**) and Occludin (**C**) in the perihematomal area at 24 h after W-ICH. The arrow indicates the breakdown of the continuous endothelial cell layer. Scale bar = 20 µm. Representative bands and relative density analyses of the ZO-1 (**B**) and Occludin (**D**) expression in the perihematomal area at 24 h after W-ICH (*n* = 6). Data are shown as mean ± SD. *P < 0.05; **P < 0.01

Representative images of endothelial marker vWF (von Willebrand factor) co-staining with ZO-1 (tight junction-related protein) and Occludin (tight junction-related protein) revealed that GLC pretreatment protected BBB from destruction at 24 h after W-ICH (Fig. 5A, C). Similarly, WB assays demonstrated the same trends. Both the content of ZO-1 (sham 0.50 ± 0.02 vs vehicle 0.26 ± 0.08, *P* < 0.01; Fig. 5B) and Occluding (sham 0.49 ± 0.06 vs vehicle 0.26 ± 0.10, *P* < 0.01; Fig. 5D) decreased following W-ICH. However, GLC pretreatment significantly increased the content of ZO-1 compared to the vehicle group (0.39 ± 0.06 vs 0.26 ± 0.08, *P* < 0.05; Fig. 5B) and Occluding (GLC 0.42 ± 0.07 vs vehicle 0.26 ± 0.10, *P* < 0.05; Fig. 5D). These data demonstrated that GLC pretreatment evidently prevented BBB from destruction following W-ICH.

### GLC pretreatment downregulated matrix metallopeptidase 9 expression following W-ICH

**Fig. 6:**
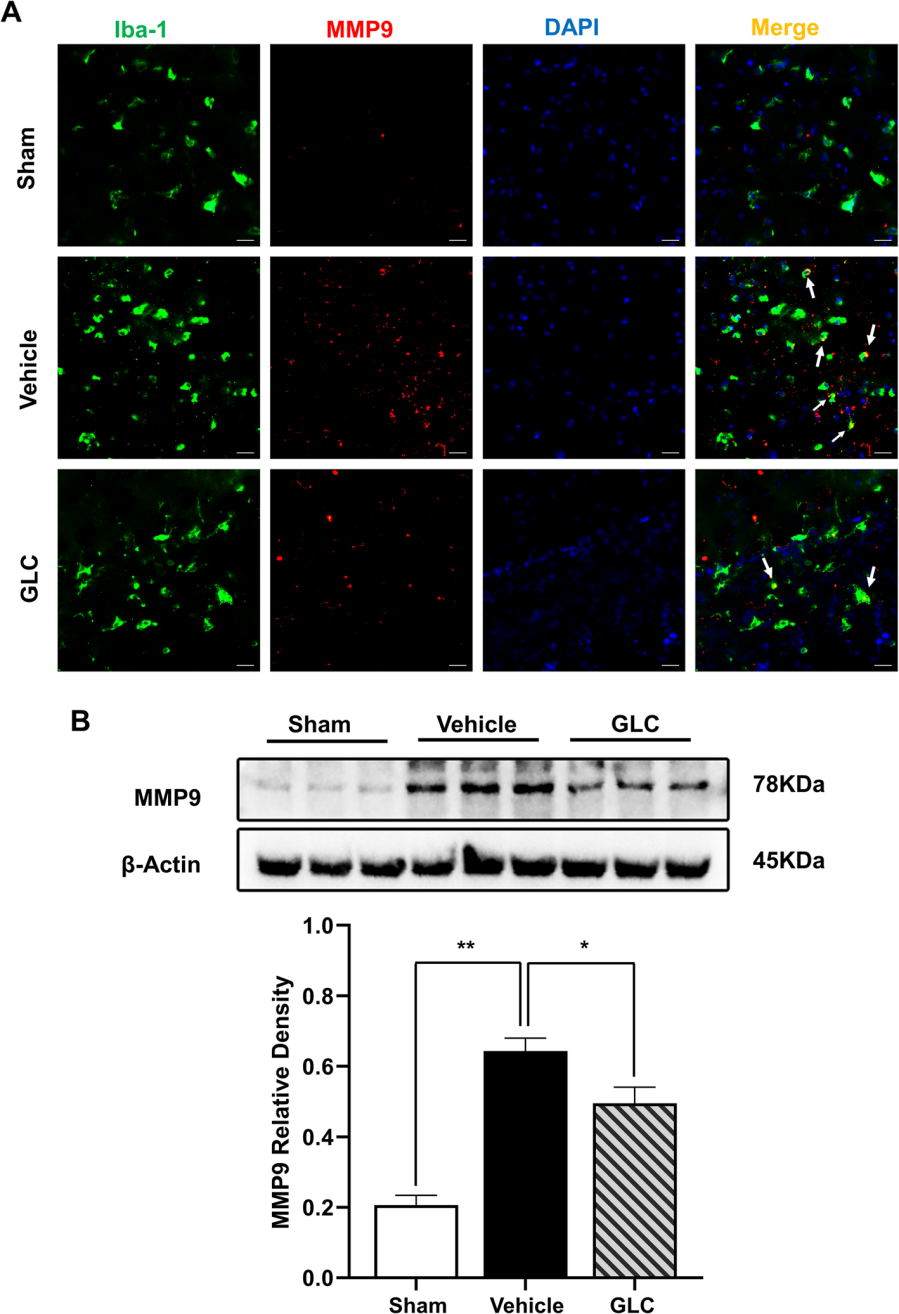
GLC pretreatment downregulated MMP9 expression following W-ICH. **A** Representative confocal images of microglial cell and MMP9.Scale bar = 20 µm. **B** Representative bands and relative density analyses of the MMP9 expression in the perihematomal area at 24 h after W-ICH (*n* = 6). Data are shown as mean ± SD. *P < 0.05; **P < 0.01

The expression of matrix metallopeptidase 9 (MMP9) at 24 h after W-ICH was examined using immunofluorescence and WB. Representative images of microglia marker Iba-1 co-staining with MMP9 revealed that W-ICH upregulated MMP9 expression, which was significantly downregulated by GLC pretreatment (Fig. 6A). In accordance with immunofluorescence, WB analysis also demonstrated that GLC pretreatment significantly prevented the upregulation of MMP9 expression induced by W-ICH (GLC 0.49 ± 0.05 vs vehicle 0.64 ± 0.04, *P* < 0.05; Fig. 6B).

## Discussion

In the present study (Supplemental Fig. 1), we demonstrated that GLC pretreatment significantly attenuated early hematoma expansion after W-ICH in rats, thus mitigating brain edema and neuronal death and promoting neurological function recovery, which maybe partially benefit from alleviating perihematomal BBB disruption by suppressing MMP9.

Hematoma expansion is a recognized predictor of poor outcome and higher mortality in ICH [19]; therefore, physicians have tried to identify hematomas that will expand in order to early intervention [20], such as spot sign on computed tomography angiography and swirl sign and island sign on non-contrast computed tomography [21]. A series of risk factors are considered to contribute to hematoma expansion [20], among which coagulation disorder is the important one [22]. This explained the result we observed that the hematoma volume of W-ICH was much larger compared with normal ICH, which contributed to higher mortality and worse outcome. Thus, combined with previous studies, restricting hematoma expansion in the early stage is a promising therapeutic approach for W-ICH [23, 24].

GLC is a second-generation sulfonylurea medication that inhibits sulfonylurea receptor 1 (Sur1) to adjust blood glucose [25]. Subsequently, GLC was demonstrated to reduce brain edema in both ischemic and hemorrhagic stroke via Sur1-transient receptor potential melastatin 4 (Sur1-Trpm4) [12, 26]. However, in recent studies, there are still controversies whether GLC could alleviate BBB damage, brain edema, and hematoma expansion in a collagenase-induced ICH [27–29]. The authors ascribed the failure to model differences, as damage in collagenase-induced ICH is more disruptive and complex than autologous whole blood-induced ICH [27, 30]. However, this explanation is insufficient, as recent clinical studies have demonstrated sulfonylurea, including GLC, benefit ICH patients [9, 31]. The point of concern in these two studies is that the authors compared diabetic patients with sulfonylurea pretreatment to diabetic patients without sulfonylurea pretreatment [9, 31]. Thus, we speculated whether GLC pretreatment could alleviate ICH, as the large majority of hematoma expansion occurred in the early stage after ICH, but it takes time for GLC to reach effective concentrations in the brain [1]. To test this hypothesis, we performed GLC pretreatment in a rat model of W-ICH. As hyperglycemia was reported to aggravate hematoma expansion in ICH [32], we chose the most commonly used dose of GLC that has no impact on blood glucose levels [27]. As expected, our results demonstrated that GLC pretreatment significantly attenuated hematoma expansion after W-ICH. Meanwhile, we evaluated the effects of GLC pretreatment on brain edema, neuronal death, and functional recovery. In agreement with our previous results, GLC pretreatment significantly reduced brain edema and neuronal death and promoted function recovery. These findings support our hypothesis that it is the timing of administration that affects the effect of GLC on ICH.

BBB disruption is one of the main mechanisms of peri-hematoma edema after ICH, and GLC was reported to reduce brain edema by preventing BBB disruption in various central nervous system injuries [12, 26, 33, 34]. So, we evaluated BBB integrity to further investigate the underlying mechanism of GLC pretreatment on W-ICH and found that GLC pretreatment prevented tight junction protein attenuation, thus reducing BBB destruction. Studies have shown that the neuroprotective effect of selective SUR1 inhibitor GLC may be partially mediated by inhibiting the expression of MMP9 [12, 35]. Blocking SUR1 can reduce the expression of MMP9 induced by ICH [12]. The result suggests that SUR1 may be closely related to the expression of MMP9 after ICH. Similarly, we found the expression of MMP9 increased after W-ICH but was significantly suppressed by GLC pretreatment. Therefore, there is reason to believe that GLC has an indirect inhibitory effect on MMP9 by inhibiting Sur1. Moreover, MMP9 was proven to contribute to BBB destruction after ICH [12, 18]. Activated MMP9 degrades the neurovascular matrix by digesting the main components of the basal layer around blood vessels (type IV collagen, laminin, and fibronectin) and the proteins (such as occludins and claudin-5) forming the tight junctions [36, 37]. Excessive proteolytic activity leads to vascular wall damage, loss of vascular integrity, and increased permeability, thereby promoting the destruction of BBB [38]. As mentioned earlier, we believe that the protective effect of GLC may be related to the inhibition of MMP9. However, we need more research to explore the mechanism of SUR1 and MMP 9 after W-ICH.

As our present study was designed to obtain proof of concept, there are some limitations. Firstly, the dose of GLC was chosen based on previous studies, and this commonly used dose was proven to have little impact on blood glucose [12, 27]. More doses and administration schedules should be designed to explore better effects. Secondly, we just focused on the effect of GLC pretreatment on the acute phase of W-ICH, and long-term outcomes should be evaluated in future studies. Finally, as an important component of the central nervous system injury response, although our study suggests that the selective SUR1 inhibitor GLC may exert a protective effect on BBB by inhibiting the expression of MMP9, the precise mechanism between SUR1 and MMP9 deserves further exploration in future studies.

## Conclusion

Our present study demonstrated that GLC pretreatment significantly attenuated early hematoma expansion, mitigated secondary injury, and improved functional recovery after W-ICH. Moreover, our data also demonstrated that GLC pretreatment prevented BBB disruption via suppressing MMP9 expression. Therefore, our findings provide initial proof of concept suggesting that GLC in stroke primary and secondary prevention might be a promising therapy for hematoma expansion at the early stage of W-ICH.

### Supplementary Information

**Additional file 1: Supplemental Table 1.** Blood glucose concentration at different time points after GLC treatment. **Supplemental Fig. 1.** Schematic design of the present study.

## Data Availability

All data generated or analyzed during this study are included in this published article. The datasets used and/or analyzed during the current study are available from the corresponding author on reasonable request.

## Abbreviations

ICH: Intracerebral hemorrhage
GLC: Glibenclamide
W-ICH: Warfarin-associated intracerebral hemorrhage
MRI: Magnetic resonance imaging
BWC: Brain water content
EB: Evans blue
INR: International normalized ratio
DMSO: Dimethyl sulfoxide
PBS: Phosphate-buffered solution
BBB: Blood-brain barrier
MMP9: Matrix metallopeptidase 9

## References

[1] Broderick JP, Grotta JC, Naidech AM, Steiner T, Sprigg N, Toyoda K (2021). The story of intracerebral hemorrhage. Stroke.

[2] Bowry R, Parker SA, Bratina P, Singh N, Yamal J-M, Rajan SS (2022). Hemorrhage enlargement is more frequent in the first 2 hours: a prehospital mobile stroke unit study. Stroke.

[3] Li Z, You M, Long C, Bi R, Xu H, He Q (2020). Hematoma expansion in intracerebral hemorrhage: an update on prediction and treatment. Front Neurol.

[4] Aguilar MI, Hart RG, Kase CS, Freeman WD, Hoeben BJ, García RC (2007). Treatment of warfarin-associated intracerebral hemorrhage: literature review and expert opinion. Mayo Clin Proc.

[5] Quintas S, Zapata-Wainberg G, Arias-Rivas S, Ximénez-Carrillo Á, Castillo J, Benavente Fernández L (2021). Time trends in intracerebral hemorrhage associated with oral anticoagulation and its risks factors in Spain from 2008 to 2015. Eur Neurol.

[6] Cervera Á, Amaro S, Chamorro Á (2011). Oral anticoagulant-associated intracerebral hemorrhage. J Neurol.

[7] Yan Z, Chen S, Xue T, Wu X, Song Z, Wang Z (2021). The function of tranexamic acid to prevent hematoma expansion after intracerebral hemorrhage: a systematic review and meta-analysis from randomized controlled trials. Front Neurol.

[8] Jiang B, Zhang Y, Wang Y, Li Z, Chen Q, Tang J (2021). Glibenclamide attenuates neuroinflammation and promotes neurological recovery after intracerebral hemorrhage in aged rats. Frontiers in Aging Neuroscience.

[9] Irvine H, Male S, Robertson J, Bell C, Bentho O, Streib C (2019). Reduced intracerebral hemorrhage and perihematomal edema volumes in diabetics on sulfonylureas. Stroke.

[10] Zhao J, Song C, Li D, Yang X, Yu L, Wang K (2022). Efficacy and safety of glibenclamide therapy after intracerebral haemorrhage (GATE-ICH): a multicentre, prospective, randomised, controlled, open-label, blinded-endpoint, phase 2 clinical trial. eClinicalMedicine.

[11] Foerch C, Arai K, Jin G, Park K-P, Pallast S, van Leyen K (2008). Experimental model of warfarin-associated intracerebral hemorrhage. Stroke.

[12] Jiang B, Li L, Chen Q, Tao Y, Yang L, Zhang B (2016). Role of glibenclamide in brain injury after intracerebral hemorrhage. Transl Stroke Res.

[13] Tan Q, Li Y, Guo P, Zhou J, Jiang Z, Liu X (2019). Tolvaptan attenuated brain edema in experimental intracerebral hemorrhage. Brain Res.

[14] Chen Q, Shi X, Tan Q, Feng Z, Wang Y, Yuan Q (2017). Simvastatin promotes hematoma absorption and reduces hydrocephalus following intraventricular hemorrhage in part by upregulating CD36. Transl Stroke Res.

[15] Tan Q, Guo P, Zhou J, Zhang J, Zhang B, Lan C (2019). Targeting neutrophil extracellular traps enhanced tPA fibrinolysis for experimental intracerebral hemorrhage. Transl Res.

[16] MacLellan CL, Girgis J, Colbourne F (2016). Delayed onset of prolonged hypothermia improves outcome after intracerebral hemorrhage in rats. J Cereb Blood Flow Metab.

[17] Feng Z, Tan Q, Tang J, Li L, Tao Y, Chen Y (2017). Intraventricular administration of urokinase as a novel therapeutic approach for communicating hydrocephalus. Transl Res.

[18] Tan Q, Chen Q, Niu Y, Feng Z, Li L, Tao Y (2017). Urokinase, a promising candidate for fibrinolytic therapy for intracerebral hemorrhage. J Neurosurg.

[19] Delcourt C, Huang Y, Arima H, Chalmers J, Davis SM, Heeley EL (2012). Hematoma growth and outcomes in intracerebral hemorrhage: the INTERACT1 study. Neurology.

[20] Caplan LR (2016). Recognizing and preventing intracerebral hematoma expansion. JAMA Neurol.

[21] Morotti A, Boulouis G, Dowlatshahi D, Li Q, Shamy M, Salman RA-S (2023). Intracerebral haemorrhage expansion: definitions, predictors, and prevention. Lancet Neurol..

[22] Burchell SR, Tang J, Zhang JH (2017). Hematoma expansion following intracerebral hemorrhage: mechanisms targeting the coagulation cascade and platelet activation. Curr Drug Targets.

[23] Flibotte JJ, Hagan N, O’Donnell J, Greenberg SM, Rosand J (2004). Warfarin, hematoma expansion, and outcome of intracerebral hemorrhage. Neurology.

[24] Schlunk F, Van Cott EM, Hayakawa K, Pfeilschifter W, Lo EH, Foerch C (2012). Recombinant activated coagulation factor VII and prothrombin complex concentrates are equally effective in reducing hematoma volume in experimental warfarin-associated intracerebral hemorrhage. Stroke.

[25] Aittoniemi J, Fotinou C, Craig TJ, de Wet H, Proks P, Ashcroft FM (2008). SUR1: a unique ATP-binding cassette protein that functions as an ion channel regulator. Philos Trans R Soc B Biol Sci.

[26] King ZA, Sheth KN, Kimberly WT, Simard JM (2018). Profile of intravenous glyburide for the prevention of cerebral edema following large hemispheric infarction: evidence to date. Drug Des Dev Ther.

[27] Arai K, Wilkinson CM, Brar PS, Balay CJ, Colbourne F (2019). Glibenclamide, a Sur1-Trpm4 antagonist, does not improve outcome after collagenase-induced intracerebral hemorrhage. PLoS One.

[28] Karamyan V, Kung TFC, Wilkinson CM, Dirks CA, Jickling GC, Colbourne F (2021). Glibenclamide does not improve outcome following severe collagenase-induced intracerebral hemorrhage in rats. PLoS One.

[29] Shiokawa R, Otani N, Kajimoto R, Igarashi T, Moro N, Suma T (2022). Glibenclamide attenuates brain edema associated with microglia activation after intracerebral hemorrhage. Neurochirurgie.

[30] MacLellan CL, Silasi G, Poon CC, Edmundson CL, Buist R, Peeling J (2007). Intracerebral hemorrhage models in rat: comparing collagenase to blood infusion. J Cereb Blood Flow Metab.

[31] Chang JJ, Khorchid Y, Kerro A, Burgess LG, Goyal N, Alexandrov AW (2017). Sulfonylurea drug pretreatment and functional outcome in diabetic patients with acute intracerebral hemorrhage. J Neurol Sci.

[32] Liu J, Gao B-B, Clermont AC, Blair P, Chilcote TJ, Sinha S (2011). Hyperglycemia-induced cerebral hematoma expansion is mediated by plasma kallikrein. Nat Med.

[33] Simard JM, Sheth KN, Kimberly WT, Stern BJ, del Zoppo GJ, Jacobson S (2013). Glibenclamide in cerebral ischemia and stroke. Neurocrit Care.

[34] Simard JM, Geng Z, Woo SK, Ivanova S, Tosun C, Melnichenko L (2008). Glibenclamide reduces inflammation, vasogenic edema, and caspase-3 activation after subarachnoid hemorrhage. J Cereb Blood Flow Metab.

[35] Simard JM (2012). Does inhibiting Sur1 complement rt-PA in cerebral ischemia?. Ann N Y Acad Sci.

[36] Rosenberg GA (2002). Matrix metalloproteinases in neuroinflammation. Glia.

[37] Cauwe B, Opdenakker G (2010). Intracellular substrate cleavage: a novel dimension in the biochemistry, biology and pathology of matrix metalloproteinases. Crit Rev Biochem Mol Biol.

[38] Gurney KJ, Estrada EY, Rosenberg GA (2006). Blood-brain barrier disruption by stromelysin-1 facilitates neutrophil infiltration in neuroinflammation. Neurobiol Dis.

