# Supplementary material for: Glibenclamide pretreatment attenuates early hematoma expansion of warfarin-associated intracerebral hemorrhage in rats by alleviating perihematomal blood–brain barrier dysfunction
Source: Chin Neurosurg J. 2023 Dec 7;9:35. doi: 10.1186/s41016-023-00351-2 (PMC10701923; doi:10.1186/s41016-023-00351-2)

**Supplemental Materials**

**Supplemental Table 1. Blood glucose concentration at different time points after GLC treatment.**

| Time after GLC treatment | Vehicle | GLC | p value |
| --- | --- | --- | --- |
| Before | 7.92 ± 0.76 | 8.05 ± 1.09 | 0.46 |
| Day1 | 7.85 ± 0.64 | 7.70 ± 0.79 | 0.65 |
| Day2 | 7.52 ± 0.49 | 7.40 ± 0.76 | 0.36 |

Data are expressed as mean ± SD; GLC: Glibenclamide.

**Supplemental Figure 1.** Schematic design of the present study.


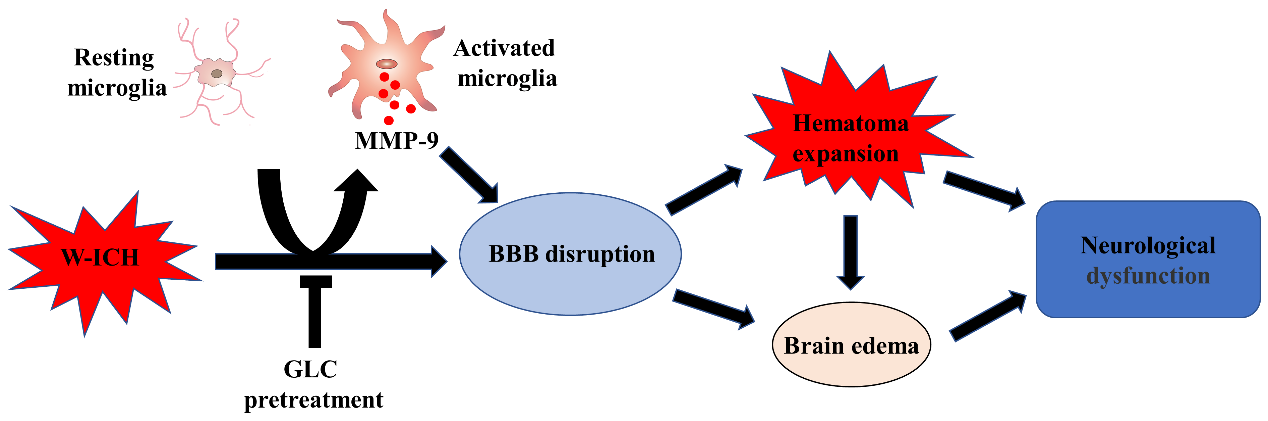

Supplement: Supplementary file 1 — Additional file 1: Supplemental Table 1. Blood glucose concentration at different time points after GLC treatment. Supplemental Fig. 1. Schematic design of the present study. [file 41016_2023_351_MOESM1_ESM.docx]
